# A science communication-focused summer project boosts first year bioscience students’ skill gains and supports placement year uptake

**DOI:** 10.1016/j.crphys.2025.100155

**Published:** 2025-07-04

**Authors:** Vanessa L. Armstrong, Beth M. Lawry, Harley J. Stevenson-Cocks

**Affiliations:** School of Biomedical, Nutritional and Sport Sciences, Faculty of Medical Sciences, Newcastle University, Newcastle upon Tyne, UK

**Keywords:** Science communication, Placement year, Employability, Transferable skills

## Abstract

Communication skills are an essential transferable skill for graduates and, for bioscience students, science communication skills are fundamental to achieving success. Increasingly, development of enhanced wider transferable skills, often outside of normal bioscience curricula, is required during university study for graduates to achieve positive outcomes amongst an increasingly competitive job market. Innovative approaches to improve student development, confidence and skill gain are therefore required to maintain positive graduate outcomes.

Using science communication as a focal point, a novel three-week summer project was developed to provide a platform for first year bioscience students’ skill gain, with a view to enhance participants’ employability particularly for attaining competitive industrial placement year positions. The project was delivered entirely remotely via Zoom and Microsoft Teams and required participants to complete three communication-focused assessments centred on recent papers highlighted by the University’s Press Office. The pedagogical impact of this summer project was evaluated through pre- and post-project surveys from four iterations of the project (2021–2024) focusing on participants’ self-evaluation of skills aligned to the University’s Graduate Framework.

To date, 89 Level 4 bioscience students at a research-intensive UK university (and its Malaysian sister campus, n = 8) have completed the project. Project participants primarily wanted to improve their Academic Writing (93 %), Communication (85 %) and Research (89 %) skills as well as their Critical Thinking (72 %), Teamwork (74 %), Collaboration (59 %) skills and Confidence (68 %). Post-project, significant increases in 16 of 18 skills were reported by participants (*P* < 0.05). Of 29 participants that wanted to secure a placement year, 21 (72 %) went on to do so and a longitudinal survey of these participants (n = 16 respondents) revealed they evidenced the project in their applications (100 %, n = 16) and 75 % (n = 12) were specifically asked about the project at the interview stage, using the project as evidence of transferable skill development.

The project has demonstrated strong potential to boost participants’ skill development and employability, while providing a platform for academic improvement and transnational engagement. With a simple focus on communication skills and an accessible, adaptable format, the project provides a framework for other institutions to utilise to enhance student outcomes in the biosciences and beyond.

## Highlights

• A communication-focused summer project improves student skill development.
• Enhanced skills and confidence contribute to greater placement year attainment.
• A flexible and accessible format provides adaptability for other institutions.
• Transnational education and institutional awareness enhanced by project completion.

## 1 Introduction

Effective communication is a key transferable skill that all graduates across Higher Education need to develop to succeed whilst studying, in securing competitive positions such as placement/sandwich years, and post-graduation when entering the ever-evolving workplace (Hill et al., 2016; Oliver et al., 2018). The type of communication and target audience varies across disciplines, but for life sciences the importance of effective science communication has never been greater given the rise in social media usage, misinformation, and ability to reach larger audiences with global platforms now being the default (Baram-Tsabari et al., 2017; Goldstein et al., 2020).

Many Higher Education Institutions (HEIs) include communication alongside other transferable skills in their list of graduate attributes – the skills, beyond discipline-specific knowledge, each HEI expects their graduates to achieve from within the curriculum or via the extracurricular offer (Barrie, 2012). As well as communication, these skills include teamwork, organisation, time management, critical thinking, adaptability, resilience and more, and there were concerns dating back to the 1990s that graduates were ill-equipped for the job market by not sufficiently developing these (Foley, 1999). Ensuring graduates have the appropriate skills for employment is essential and the reported skills gap between graduates and the global demand for highly skilled individuals means there is a pressure for HEIs to effectively deliver and create opportunities for skill development (Department for Education, 2023; European Commission, 2017). Metrics such as the Teaching Excellence Framework and National Student Survey measure how successful HEIs are in terms of their skill development provision, but students also recognise the development and importance of transferable skills across their studies and it is essential that this starts in year 1 and that time and space are allocated towards this in the curriculum (Haigh et al., 1999).

Creating opportunities where students can work collaboratively in a low-risk environment in smaller groups has been shown to be beneficial to the student experience (Francis et al., 2025) and skills such as teamwork are recognised as important by key stakeholders (employers, staff and students) (Haigh et al., 1999; Francis et al., 2025), however doing so at scale when undergraduate cohort sizes are large is a major challenge to HEIs. Therefore, innovative approaches to providing opportunities for student skill development outside of the curriculum are needed.

The benefits of students undertaking extracurricular activities during their undergraduate studies are well documented and include development of transferable skills, improving sense of belonging, and improved academic performance (Buckley et al., 2021; Christison, 2013; Fakhretdinova et al., 2021). Official recognition of participation in these activities, such as through the Higher Education Achievement Record, achieving an award, certification or digital badge provision allows students to easily demonstrate their extracurricular involvements and potentially help them seek successful graduate-level destinations and allow students to stand out from their peers in a highly competitive employment market. However, not all students are able to partake in these activities due to other commitments such as balancing part-time work, caring responsibilities and their geographical location (Dickinson et al., 2021). Online provision and utilisation of digital platforms and recordings has been shown to overcome some of these barriers (Baker et al., 2020; Hewson et al., 2021) and support student engagement and inclusivity.

The most formalised and substantial experience a student can attain during their university study is a placement or sandwich year in which students take a year out from their studies and work full-time in industry. These experiences greatly enhance students’ personal and professional development and have been shown to improve subsequent academic performance (Brooks et al., 2016; Gomez et al., 2004; Hejmadi et al., 2012; Reddy et al., 2006, Reddy et al., 2012). Undertaking a placement year has also been shown to reduce the awarding gap in comparison to non-placement year students, but not all students engage with this opportunity with some groups of students being underrepresented (Traynor et al., 2024).

These 9–12-month roles are highly competitive and often involve multi-stage application processes. Within the Biomedical and Biomolecular Sciences degrees at Newcastle University, approximately 8–10 % of students secure and complete this type of opportunity between year 2 and 3 of their undergraduate degree. The need to stand out from the crowd and have relevant experiences to reflect upon within the application process is key to success in achieving these positions, alongside tailored support with the complex application process (Shanmugam et al., 2024). Across the UK, there has been an increase in the number of degrees that offer placement years as an option, recognising the benefits of undertaking work-based learning and other initiatives by HEIs over the past 20 years to promote student employability (Little et al., 2007).

Employers value communication skills highly when recruiting students and graduates (Coffelt et al., 2019) but there is a gap between employers’ expectations and graduates’ perceptions of their abilities (Chan, 2024), highlighting the need for enhanced communication skill training in HEIs. Several institutions have developed specific optional science communication modules where various facets of the broad topic of science communication are covered, including different modalities for communicating science such as blogs, live projects, press releases and lay abstract writing (Yeoman et al., 2011). Embedding some of these facets into the curriculum for all students is the ideal but limited time and space make this challenging particularly with there being no defined curriculum for science communication courses (Bray et al., 2012; Lewenstein et al., 2022; Oliveira et al., 2024). Designing new programmes or curricula is often the only available avenue for including training and assessment linked to science communication, but is necessary given the increasing requirement for effective lay communication in grant criteria. Thus, enhanced provision of training for undergraduate skill development is a logical step to developing researchers of the future.

Key to effective science communication is the ability to explain complicated scientific findings to a non-specialist audience, a focus of a successful year three (FHEQ Level 6) optional Science Communication module ran since 2012 at Newcastle University (Newcastle University, 2025). Utilising experience and pedagogical insight from this module, a new extracurricular project was developed for first year bioscience students with a view to develop their transferable skills and employability using the principles of science communication as the focus point. This paper showcases the set-up, delivery and evaluation of the ‘SciComm Summer Project’ since its inception in spring 2021 when it became apparent that student opportunities for engaging in extracurricular enrichment activities would be negatively impacted by the COVID-19 pandemic, with research placements, shadowing/volunteering opportunities and placement years all reduced across the life science sector during this time.

The aim of the project was to create an opportunity that would support students both academically and with transferable skill development, and importantly create an inclusive and flexible opportunity for a range of students to engage in and help them to stand out when seeking future opportunities such as placement years and employment (Wang, 2024). By offering this opportunity to year 1 students at both Newcastle University and its Malaysian sister campus NUMed (which runs a 2 + 1 programme where students complete their final year at the Newcastle campus), it was also intended to help the integration of students across both campuses, enhance transnational communication and awareness, and improve student transition and support for those participants joining from NUMed. To evaluate the impact of the project on graduate attribute development, knowing that career management skills are often overlooked in bioscience curricula (Bridgstock, 2009), the project utilised participant self-evaluation and skills reflection pre- and post-project, with a focus on career readiness, placement year interest and subsequent attainment.

Following a successful pilot of the SciComm Summer Project in 2021, three further iterations of the project have ran to date and the utility of the project in participant skill development, and as a flexible and adaptable platform for other HEIs to consider, is reported herein.

## 2 Methods

### 2.1 Course context and participants

The SciComm Summer Project was developed as a 3-week optional course for first year undergraduate students (FHEQ Level 4) enrolled on BSc or integrated Master’s (MSci) degrees in Biochemistry, Biomedical Genetics, Biomedical Sciences, Pharmacology or Physiological Sciences (collectively termed Biomedical and Biomolecular Sciences), at a large research-intensive (Russell Group) university in the UK and its Malaysian sister campus (NUMed). The Biomedical and Biomolecular Sciences programmes at Newcastle University typically enroll approximately 350–400 students each academic year from across the world and diverse backgrounds (between 10 and 20 % are international students), with approximately 30 students joining Level 6 from NUMed. The SciComm Summer Project was open every July to all year 1 students from both campuses.

**Fig. 1:**
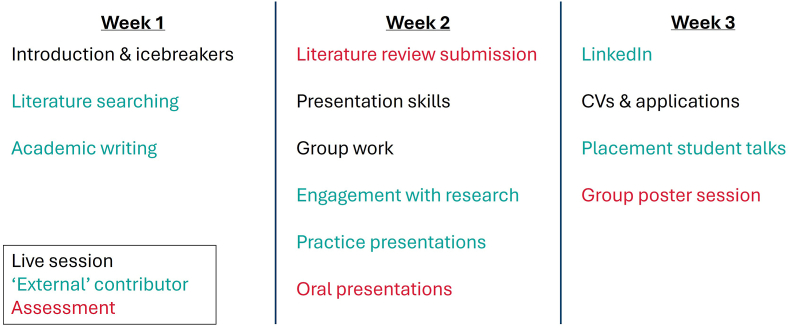
A typical timetable for the SciComm Summer Project. A normal iteration of the project totaled 20–25 h’ worth of contact time, delivered by a mix of School academic staff and contributors from other University departments (denoted by ‘External’ contributor).

The course was advertised each year for students to apply to by submitting a CV and short statement of motivation (<200 words) towards the end of their first year of study. Taught elements of the course were delivered as synchronous online sessions via Zoom, with participant work otherwise conducted asynchronously. All sessions were recorded and recordings made available to participants immediately afterwards to not discourage participation from those undertaking part-time work, internships, with caring responsibilities or otherwise unable to attend live (e.g. due to time zone differences for international students). A typical iteration of the project comprised approximately 15–20 h of contact time (Fig. 1) over a 3-week period and the project team set an upper limit of 30 participants to manage workload and facilitate effective teamwork throughout. All applicants were accepted onto the project unless the limit was reached, or the applicant’s submission was insufficient (neither of which had occurred to date).

### 2.2 Course design

Project participants were split into small groups (maximum 6 participants) with each group assigned a recent scientific paper involving Newcastle University researchers, chosen each year from a selection of bioscience papers recently highlighted by the University’s Press Office. Participants were added to a dedicated Team on Microsoft Teams which hosted resources, communication and information relating to the project. Microsoft Teams was chosen rather than the University’s Virtual Learning Environment (VLE), Canvas, to further enhance participants’ digital skills and awareness of collaborative platforms. Live sessions were delivered via Zoom using the recurring meeting function to streamline recordings and for ease of access for participants.

Over the 3-week project period, participants were trained in and completed three assessments comprising an individual literature review pitched at a typical academic audience, an individual oral presentation pitched at GCSE-age (15–16 years’ old) students, and a group poster presentation in a conference-style virtual poster session pitched at undergraduate student peers. Skills support sessions including literature review writing, presentation skills and group work, as well as supplementary sessions including engagement with research, CV/LinkedIn profile development, and past/present placement student talks were delivered by academic staff and other ‘external’ contributors from across the University, including from the University’s Careers Service, Engagement and Outreach, Academic Skills and 10.13039/100005721Enterprise teams. Engaging contributors from across the University helped to ease the project team’s workload, while also gaining access to wider expertise from colleagues and enhancing the exposure of their respective teams amongst the student cohort.

Participant assessments were completed weekly throughout the project, though flexibility was offered on deadlines where participants may have had exceptional or unique individual circumstances (e.g. due to part-time work or other commitments) to not discourage engagement. To streamline academic workload and marking burden, simple standardised feedback was provided by the academic project team in the form of ‘3 good things’ and ‘3 things to improve’ for each assessment. At the time of project development, this also aligned with the School’s strategy to adopt more consistent feedback practice and mirrored the approach participants were already familiar with from their undergraduate coursework.

Importantly, throughout the project, emphasis was placed on participants developing their wider skill portfolio and supporting their future employability. To achieve this, the University’s Graduate Framework was adopted and skills therein were regularly signposted to participants to encourage their self-reflection and skills development. Two anonymous evaluation surveys were also designed and introduced to not only evaluate participants’ perceptions of the course but also to help facilitate reflection on the skills they wished to develop and then did go on to develop following project completion.

Three distinct Learning Outcomes (LOs) were developed, aligned to the project’s main goals of communication and transferable skill development, to aid students’ contextualised learning. These were that, at the end of the project, students would be able to:

• LO1: Explain and summarise findings from a research paper to different target audiences.
• LO2: Apply a range of communication modalities working both independently and within a team.
• LO3: Recognise the importance of communication and transferable skills to ensure they are future fit for employability and their studies.

Lastly, to recognise participant completion of the project, a digital badge was developed for participants to claim via https://openbadges.org/ (1EdTech) and use to strengthen their CVs and LinkedIn profiles aligned to LO3 above (Dowling-Hetherington et al., 2017).

### 2.3 Evaluation surveys

The project was evaluated each year through participants completing anonymous pre- and post-project surveys, to investigate their academic and graduate framework skill development as well as to evaluate the delivery of the project overall. Both surveys were designed to be brief, utilising interval scales for skill rating (1–5, representing a Likert scale of Very Poor to Very Good) in order to maximise completion and ensure participants did not rush through the survey (McBean et al., 1982). Participants were briefed on the purpose of the surveys and their right not to complete, or subsequently withdraw, as part of obtaining consent to participate in the study.

Pre- and post-project surveys were delivered separately via SurveyMonkey (SurveyMonkey Inc.) before the start (to gather baseline data on participants’ self-reported skills) and at the end of the project (to determine the impact of the project), respectively. Responses to both surveys were linked using a numerical identifier that still maintained participant anonymity. Questions aligned to skill development were worded identically in both surveys and focused on core academic skills linked to the university’s Graduate Framework. The pre-project survey also explored other career readiness factors such as desire to pursue a placement year, participation in extracurricular activities or part-time work, and whether participants knew their post-university career plans. The post-project survey further investigated participants’ desire to pursue a placement year (to see if the project had altered their plans) and evaluated the project’s delivery through a series of 5-point Likert-style and yes/no questions (some with an option for “don’t know” or “unsure” to allow ambivalence (Wright et al., 2010)) for the project team to refine ahead of subsequent iterations.

### 2.4 Placement catch-up survey

As an initial aim of the project was to provide experience for participants to evidence in their subsequent applications for industrial placement years, a further anonymous follow-up survey was designed for participants that completed the course and went on to secure placement year positions. This survey was also administered via SurveyMonkey and asked participants to self-report on their strength in the same skills as before, whilst also exploring whether the project provided academic benefit in their second-year studies, if and how they mentioned the project in their interviews and applications for placement year positions, and if so what elements of the project were mentioned or discussed.

### 2.5 Statistics/data analysis

Survey data was analysed using Microsoft Excel and survey data are reported as mean ± standard deviation. Paired Student’s t-tests were performed to determine significant differences between pre- and post-project responses. Statistical significance was determined at *P* < 0.05 for all statistical analyses.

## 3 Results

### 3.1 Participant demographics

Since the first project cohort in 2021, 89 Level 4 students have successfully completed the project; including 72 females and 17 males, which is a higher female ratio than the representative degree cohort; 55 Home UK, 26 international students and 8 students from NUMed. The percentage of international students on this project is greater, 29 % (38 % inclusive of NUMed students), than the overall biosciences Level 4 cohort (12 %).

### 3.2 Prior experiences of participants

**Fig. 2:**
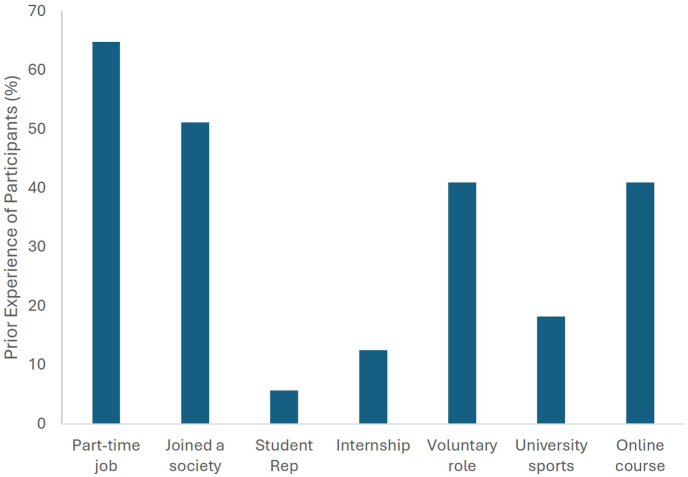
Exploring the extracurricular experience of participants prior to the project, displayed as percentage of total respondents (n = 88). Participants could select more than one experience.

There were 88 responses to the pre-project survey and respondents had a range of prior extracurricular experiences, with 65 % of survey respondents having worked part-time, and 51 % involved in university societies (Fig. 2). Voluntary roles and online courses were also listed highly with 41 % of respondents selecting these extracurricular experiences.

### 3.3 What skills did participants want to develop in the project?

**Fig. 3:**
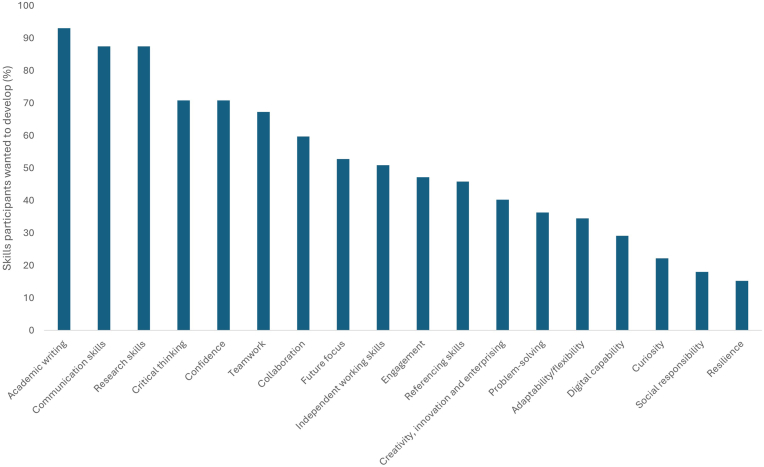
Skills that participants wanted to develop within the project, displayed as a percentage of total respondents (n = 88). Participants could select multiple skills.

The skills that participants wanted to develop during the project are shown in Fig. 3. The most frequently selected skills were ‘Academic Writing,’ ‘Communication Skills,’ and ‘Research Skills.’ In contrast, the skills with the lowest selection percentages were ‘Curiosity,’ ‘Social Responsibility,’ and ‘Resilience.’

### 3.4 How did participants rate their skills pre- and post-project?

**Fig. 4:**
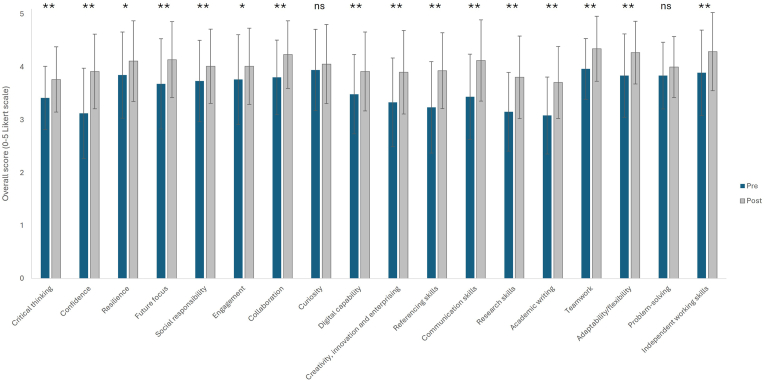
Participants transferable skills rating pre- and post-project, n = 72, ∗*P* < 0.05, ∗∗*P* < 0.01, ns = not significant (paired Student’s t-test).

To determine the impact of the project on the transferable skills of the participants, skills were self-rated pre- and post-project (Fig. 4). There were 72 respondents for the post-project survey, which showed an increase in ratings for all self-reported skills. Additionally, 16 out of the 18 skills significantly increased (*P* < 0.05), with 14 of these, including Confidence, Communication, and Teamwork, increasing with a significance of *P* < 0.01.

### 3.5 Project highlights

When asked which session was the favourite, there was a spread of answers that covered the full range of sessions provided. When asked for free text comments about the overall highlights of the project, participants again stated a range of topics including learning how to use Microsoft Teams, teamwork, experience of presenting to ‘15-year olds’, meeting new people, getting to know the lecturers and being appreciative of the recorded online sessions. When asked for improvements, some participants commented that they would prefer a general feedback session, more literature review support, more group work with changing groups, and some in-person sessions too (Table 1).

**Table 1:** Quotes from open text comments from participants when asked for the highlights and how to improve the project.

Highlight/to improve	Participant quotes post-project
Highlight	“Meeting people from my course and developing my communication and academic writing skills. I also now know how to use MS Teams correctly!”
Highlight	“Working with new people and the presentation to 15-year-olds”
Highlight	“Meeting new people on the course and getting to know the lecturers”
Highlight	“Independently working and being proactive whilst also gaining teamwork experience and communication practice.”
Highlight	“The presentation. It was so interesting to try to cater to that specific audience and the feedback was very useful.”
Highlight	“Working with a group of people I’ve never met with or talked to before”
Highlight	“We had great support and useful tips/tricks for presenting”
Highlight	“It opens my mind to the opportunities that are out there.”
Highlight	“Being able to meet people who take similar courses to me all across the world and seeing their take on how they present a topic.”
To improve	“A general feedback session regarding the literature review, or just a general project review session to end the project with.”
To improve	“I think it would be nice to have a few more group activities, perhaps switching with different people over the 3 weeks.”
To improve	“some part of the project was in person”
To improve	“It would be great to go in-depth on the literature review, more tips and tricks, things to avoid, etc.”
To improve	“present posters in-person”

### 3.6 Overall project feedback

When asked if support on the project was sufficient, 96 % (n = 69) selected ‘quite’ or ‘very sufficient’, one survey respondent selected ‘neither’, and two ‘not very’. Of the 72 respondents, 99 % (n = 71) selected ‘A bit’ or ‘A lot’ when asked if the project enhanced employability, with only one responding ‘not really’, 97 % (n = 70) said they would recommend the project to a friend and two selected ‘don’t know’, and 99 % (n = 71) of respondents reported they enjoyed the project ‘a bit’ or ‘a lot’ with only one response of ‘no opinion’. The free text comments were also overwhelmingly positive.

### 3.7 Placement attainment and project utility

**Fig. 5:**
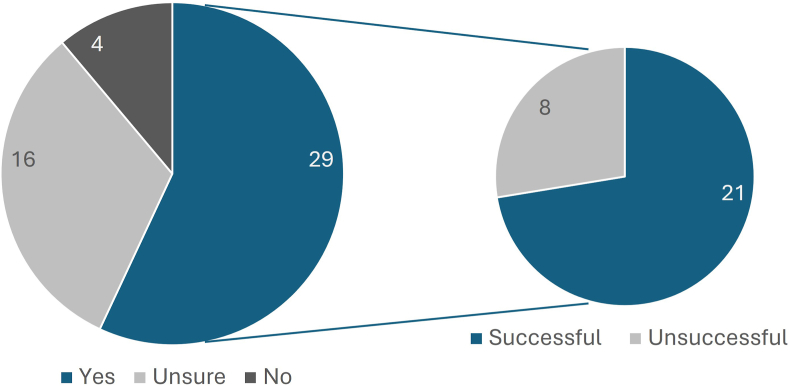
Professional placement year interest and application success in the 49 survey respondents from years 2021, 2022 and 2023. Left: breakdown of responses to the question “Are you hoping to undertake a professional placement year between stages 2 and 3?“. Right: breakdown of placement application outcomes of the 29 respondents who answered ‘Yes’ to the previous question.

The initial aim of the project was to provide experience for participants to evidence in their subsequent applications for professional placement years. Placement data is included for project cohort years 2021, 2022 and 2023 only (as 2024 participants are currently in the application stage for placement years) (Fig. 5). From these cohorts there were 49 survey respondents, 59 % of whom (n = 29) wanted to pursue a placement-year, with 33 % (n = 16) unsure and 8 % (n = 4) not interested. Of the 29 who wanted to apply for professional placements, 72 % (n = 21) were subsequently successful. The participants who successfully gained a placement year were later surveyed to investigate the importance the project had on placement applications, with 16 respondents. All respondents highlighted this project within their applications; 75 % (n = 12) were questioned about the project during interviews and 50 % (n = 8) stated that the project was very advantageous in placement applications and interviews.

## 4 Discussion

In an increasingly competitive graduate market, university graduates require a broader range of experiences and skills in order to stand out when they graduate and gain positive graduate outcomes (Ahmad Jaber and Mohammed Shah, 2024; Dinh et al., 2023; Wahab et al., 2025) and engaging in extracurricular activities is one such way students can enhance their skill sets and profile. Extracurricular activities enhance students’ confidence, self-efficacy and social ties while also developing their skills and competences associated with employability such as problem solving, communication and team-working skills (Buckley et al., 2021; Griffiths et al., 2021). This project sought to develop and evaluate the ubiquitous skill of science communication as a platform for delivering an extracurricular opportunity to boost employability and skill gain amongst first year bioscience students at a research-intensive UK university.

To date, 89 participants have completed the project over a four-year period (2021–2024). Significant increases between the start and end of the project in self-evaluated skills were reported across 16 of 18 skills, covering a range of academic and wider transferable skills including LOs 1–3 and those related to the University’s Graduate Framework (Fig. 4). Only Curiosity and Problem-solving skills were not significantly increased post-project, although both were in the lowest-ranked skills that participants wanted to develop ahead of the project (Curiosity ranked third and Problem-solving sixth least important) and still saw slight increases post-project.

Despite the academic team’s focus for the project being on building transferable skills with a view to enhance participant employability, project participants primarily took part to enhance their primary academic skillset through building their Academic Writing, Communication, and Research skills (Fig. 2). However, there was still a clear interest in building wider transferable skills aligned to LO3 such as Confidence, Teamwork and Collaboration in particular. A desire to enhance participants’ employability and confidence in collaborative tools widely used in the workplace (Abazi Chaushi et al., 2024; Anders, 2016; Montrief et al., 2021) was justification for the use of Microsoft Teams as the delivery platform for the project as opposed to the university’s native VLE, Canvas. Deliberately navigating away from Canvas as the VLE for this project forced participants to become familiar with and upskill in using team communication platforms they may encounter in a placement or graduate setting later in their careers. This also had the advantage that it was seen as something distinct from credit-bearing modules or content. Microsoft Teams has been shown to support student learning, engagement and inclusivity in higher education as has been reported by several groups since its introduction in 2017 and wider use as a result of the COVID-19 pandemic (Baker et al., 2020; Hewson et al., 2021).

The flexible nature of the project, not requiring live, synchronous attendance and being delivered exclusively online, was a positive approach and well-received by the student participants. Many participants continued to work part-time jobs, internships or other roles over the summer during the project and would not have been able to participate otherwise, but still being able to engage with the project and keep “their science minds ticking over” was appreciated.

This flexible approach to project participation also offered a novel approach for cross-institutional and multi-national participation. To date, 38 % of project participants identified as either international students or those from the University’s Malaysian sister campus, NUMed. Thus, an unintended additional benefit of the project was enhancing international collaboration between colleagues and participants and this was reflected in the post-project feedback with participant comments such as “the fact it was online made it really convenient for those of us in other parts of the world to join and improve our skills” and “it was a really fun and exciting experience … getting to know more people over at the UK campus”.

Initially a focus of the project was to fill a potential experience gap, as a result of the COVID-19 pandemic, for students seeking a professional placement year, so a longitudinal aim of the study was to investigate the utility and benefits of the project for participants that did go on to achieve placement year roles. From the three cohorts of participants that have since progressed to this stage of university study, 72 % of participants that wanted to secure a placement year (n = 21) had done so compared to approximately 10 % of the main cohort that typically go on to achieve placement year roles (unpublished data). Importantly, all of those who responded to the placement catch-up survey (n = 16) highlighted their participation in the project in their applications, 12 of these were asked specifically about the project at the interview stage for their placements, and 15 indicated their participation in the project was advantageous for their placement applications (one stated ‘it made no difference’). From the associated free-text comments, participants cited the development of key transferable skills such as Communication, Presentation, Collaboration and Teamwork as those they most evidenced at interview, particularly during open-ended competency questions, which are a key focus of placement year interview stages (Hartwell et al., 2019; Martin et al., 2008). Therefore, this shows the project provided a valuable source of experience and evidence for participants seeking placement year opportunities (LO3). Three, rather than four, years of data from the placement catch-up survey is presented above as recruitment for placement years for the most recent cohort of participants is ongoing at the time of writing – three participants from the 2024 iteration have gone on to secure placement year positions to date (data not presented as the recruitment cycle is ongoing).

Participant feedback on the project has been overwhelmingly positive to date, with all non-positive comments mainly relating to suggestions for additional sessions and assessments or further support. One recurring point of project feedback is the provision of exemplars, particularly for the literature review assessment, as participants wish to see examples before attempting their own. Provision of exemplars is known to help learners understand what ‘good’ looks like and is a core principle of good assessment and feedback (Knight et al., 2022), however the academic team felt the training sessions provided enough support for the assessment and that providing exemplars of past literature reviews may be confusing to the participants, add an additional workload burden given the papers used for the project are different each year, and not facilitate them achieving LO1 authentically. Furthermore, exemplar provision has been suggested to only be of benefit when supported by wider interactive elements (To et al., 2021) and given the project was optional and not linked to formal summative assessments this was deemed an unnecessary avenue to pursue. The other main recurring theme to participant feedback was around providing some in-person sessions or a subsequent in-person event for participants to meet their team members and practice aspects of science communication in an in-person format. However, in-person delivery would not align with the accessibility of the course and international nature of the project.

An additional benefit of the project has been the engagement of staff teams from across the University in its delivery. Since inception of the project in 2021, delivery and support of the project has been facilitated by the authors and colleagues from non-academic professional services staff across the University’s Press Office, Careers Service, Academic Skills, Engagement and Outreach, and Enterprise teams, all of whom have expressed enjoyment with delivery of the project and had recurring, invaluable involvements each year.

Through utilising science communication as the core project focus, engaging stakeholders from across the University, and drawing on recent research publicised by the University’s Press Office, the project provides an adaptable, transferable and accessible platform for other institutions to follow irrespective of subject area. Communication skills are essential for all university students to develop (Cronin et al., 2024) and so the project’s delivery can be easily adapted to meet subject-specific standards for communication skill development. University departments and professional services teams outside of individual academic units contribute greatly, but intangibly, to student performance and success (Johnson et al., 2022; Perez-Encinas and Ammigan, 2016) and yet research suggests there is a lack of awareness amongst students of such services or that they prefer to seek academic support instead (Johnson et al., 2022). By incorporating such services into the delivery of projects such as this, their profile and utilisation is raised and may enhance student outcomes (Mireku et al., 2024; Shaheen et al., 2020). Furthermore, many universities, particularly research-intensive ones such as those in the UK Russell Group, US Ivy League or Australian Group of Eight, have Press Offices or similar public relations and marketing teams which improve engagement with their activity (Vogler et al., 2020). Utilising recent publications as highlighted by these teams offers a simple route for uptake of this project format across other institutions and may also hope to raise student awareness of research activity at their own institutions.

### 4.1 Challenges

The predominant challenge in delivery of this project was time. Delivery of the project each year commenced in early July, just after the conclusion of university examinations and examination boards hence a busy time for the academic team. However, this was the most appropriate period to avoid clashing with the normal university academic year, summer examinations and the university’s August examination resit period, and preparations for the forthcoming academic year in September. Once the academic team became familiar with the project set up and administration, this time commitment was eased.

Similarly, time spent delivering the project can also present a challenge. However, this has been mitigated over several iterations of the project through onboarding of ‘external’ contributors from across the university who took on delivery of some teaching sessions, both freeing up academic staff time and introducing student participants to fresh voices and their respective teams. This balance of delivery appeared to be appreciated by the student participants, with 13/32 respondents in the post-project survey indicating their favourite session was one delivered by an ‘external’ contributor and others remarking on the benefits of getting to know the academic team on a closer level, which is not normally possible during term-time due to time constraints and the cohort size.

Also linked to the time burden of administering the project was the marking load. With three assessments across a three-week period, marking turnaround time was tight. To alleviate this, the project team implemented a simplified approach to providing feedback which was, for each assessment, to provide ‘3 good things’ and ‘3 things to improve’. Keeping feedback restricted to this approach and only providing an overall pass/fail for the project simplified the feedback and marking process and aligned with an existing School strategy to provide a consistent approach to feedback across assessments. Reducing the extent of feedback to just these two streams allowed the team to prioritise providing tangible ‘feedforward’, which is known to be an essential part of effective feedback (Higgins et al., 2001; Nicol, 2010; Voelkel et al., 2020). No free text comments from the post-project survey indicated dissatisfaction with the approach to feedback – in fact, the majority were positive including one comment that the overall highlight was “being able to write a literature review with no grade attached and get feedback” and one that “the feedback was very useful”.

### 4.2 Limitations

The use of Likert scale questions in the pre- and post-project surveys is not without limitation. Acquiescence bias, where respondents are more likely to select affirming responses, is a known limitation of Likert scale questions (Krosnick, 1999) however this phenomenon is more associated with student experience surveys than students’ self-assessment of skills (Yorke, 2009). Likert scale surveys do, however, offer greater ease and straightforwardness of use for respondents and so from a practitioner point of view are more likely to achieve greater response rates (Spooren et al., 2008) which is important given the increasing prevalence of survey fatigue amongst higher education students (Fass-Holmes, 2022). Use of participants’ self-assessment of skill ratings as in this study assumes that self-evaluation of their work is veridical (Butler, 2011), though the purpose of this project was primarily to improve participants’ skills rather than to correlate their self-evaluations with teacher-assessed ratings (Brown et al., 2013). Thus, a simple approach to survey design and implementation aided in maximising completion over a short time period while still allowing for quantification of participant skill gain.

Completion of both surveys was not a requirement for passing the project but was strongly encouraged, and completion rates of 99 % (n = 88) for the pre-project and 81 % (n = 72) for the post-project survey are high compared to typical survey completion rates which are reported to be decreasing (Zhang et al., 2017) and as low as 39.6 % for web surveys (Cook et al., 2000). However, timely completion of the pre- and post-project surveys was a challenge and not all participants submitted their pre-project surveys ahead of the project start as intended to capture pre-project skill self-assessment. Some allowance was made for this with regular completion reminders in the first few days of the project and pre-project surveys submitted after the first assessment deadline were not included for analysis – although this always related to participants who did not submit the survey at all and so no discounting of survey data was required. The completion deadline for post-project surveys was more flexible though participants were strongly encouraged to do this by the end of the third week of the project. Feedback for all assessments was not released until after the project concluded and so participants’ post-project self-assessment of skills was not influenced by their perception of their feedback which may otherwise have introduced a potential bias in their self-assessments as students are known to focus on grades and comments justifying them rather than the process of learning (Winstone et al., 2022).

Though data are presented on project participants that went on to secure placement year positions as a metric for success of the project, it must be acknowledged that previous studies have reported that high-calibre students are more likely to choose to go on placement (Duignan, 2003; Jones et al., 2017) and so may have secured them regardless of the project. It is also not possible to directly compare the success rate of SciComm Summer Project participants in securing placements compared to the overall cohort, as the academic team are only notified of successful placement applications, so the attrition rate of those that apply but are ultimately unsuccessful is unknown, and immeasurable in a cohort size of up to 400 students. Student attainment of placement year positions is at least partly dependent on prior academic achievement and so, while project participants may have upskilled in key areas, meeting the minimum grade requirement is often a threshold used to shortlist applicants thus these participants may have gone on to secure placements anyway. However, neither academic performance pre-project or achievement post-project were factors the team sought to investigate or explore as the focus was on developing an equitable, flexible and enriching experience for participants. Though an initial aim was to address a potential gap in placement year attainment amongst participants, over time the project has evolved more to address a wider transferable and academic skills gap amongst the student participants over necessarily directly preparing them for placement years, while still retaining a route for placement-focused participants to upskill. Unpublished data from the School also indicates that in each cohort a defined group of students undertake multiple offered extra-curricular activities and so have an extensive range of experiences to add to their portfolio.

It was also not an initial aim of the project to develop a specific intervention either targeting certain demographics (e.g. those underrepresented in STEM) or exploring demographic-related differences (e.g. gender identity, Home vs International students). While some demographic data was collected in this project, this was only used to understand participants’ demographic on entry to the project and was not utilised throughout the analyses stages so as to maintain student anonymity. Therefore, while there was a higher percentage of international students undertaking the project compared to the typical bioscience cohort at Newcastle (29 % vs 12 %, or 38 % vs 12 % including NUMed students) and a higher female-to-male ratio of participants, this was not explored further as the project focus was on general skill development. Future iterations or variations of the project could, with suitable ethical approval in place, explore the differential impact of the project on skill development between different demographics, or approaches to increase uptake of this and similar projects amongst underrepresented groups of students.

Lastly, it is acknowledged that tracking subsequent academic achievement or learning gain could provide interesting additional insight into the benefits of the project beyond transferable skill development and placement year attainment rates. However, these aspects were not considered important in the development of the project and would require additional and extensive ethical approval to be able to identify individual participants in subsequent years and/or their degree classifications at graduation. Furthermore, with at least two years’ additional learning experiences by that point (for a typical 3-year BSc Honours degree) it would be difficult to correlate any increases in achievement or learning gain to this project over a multitude of other confounding factors.

## 5 Conclusion

The implementation of a short, communication-focused summer project for first year bioscience students has demonstrated significant potential in improving participants’ academic and transferable skills, and notably in relation to achievement of highly competitive placement year positions in subsequent years of study. Additional benefits of the project include enhanced cross-institutional and transnational engagement of participants and colleagues and increased visibility and engagement of wider university teams and departments within an institution. By adopting a flexible and accessible approach to delivery, the project provides a simple and adaptable format for educators across the globe to utilise to improve their own students’ skill gain and employability irrespective of subject area.

## CRediT authorship contribution statement

**Vanessa L. Armstrong:** Conceptualization, Methodology, Formal analysis, Investigation, Resources, Data curation, Writing – original draft, Writing – review & editing, Project administration. **Beth M. Lawry:** Methodology, Formal analysis, Investigation, Resources, Data curation, Writing – original draft, Writing – review & editing, Visualization, Project administration. **Harley J. Stevenson-Cocks:** Conceptualization, Methodology, Formal analysis, Investigation, Resources, Data curation, Writing – original draft, Writing – review & editing, Visualization, Project administration.

## Data

Data will be made available on request to the corresponding author. An annotated toolkit for educators looking to adopt the project is available at https://doi.org/10.25405/data.ncl.29097980.

## Ethical approval

The collection of participant survey data was reviewed and approved by the ethics committee at Newcastle University under approval references 13442/2020 (for the first iteration of the project) and 24008/2022 (for subsequent iterations). Survey data was analysed anonymously, and student participants consented to participate and were allowed to withdraw at any time.

## Funding

The authors acknowledge and thank Newcastle University’s 10.13039/501100010301Faculty of Medical Sciences Education Research, Development and Practice (ERDP) grant scheme which part-funded aspects of this project.

## Declaration of competing interest

The authors declare that they have no known competing financial interests or personal relationships that could have appeared to influence the work reported in this paper.

## Data Availability

A data availability statement is included in the revised manuscript attached
